# An Investigation of Multilingual Domestic University Student Perceptions of English for Academic Purposes

**DOI:** 10.3390/bs12090328

**Published:** 2022-09-11

**Authors:** Wenjin Vikki Bo, Lyndon Lim, Yuna Lin

**Affiliations:** Teaching & Learning Centre, Singapore University of Social Sciences, Singapore 599494, Singapore

**Keywords:** English for academic purposes, learning experiences, higher education, multilingual students

## Abstract

It is well-established that English for academic purposes (EAP) programmes are critical for academic success in English-medium universities. Nonetheless, there is significantly less research on how EAP programmes impact multilingual domestic university students, compared to that of international students who speak English as a foreign language. While an earlier study on a university in Singapore found that an EAP programme had a statistically significant and positive intervention effect on students’ grade point average of the first semester upon matriculation, this study sought to investigate the perceptions of students from the same university, as this would contribute to how EAP programmes could be refined to better support learning. Semi-structured interviews were conducted with 50 students invited based on a maximum variation strategy. Based on the thematic analyses undertaken, four themes (i.e., programme delivery, linguistic improvement, learning transfer and change in self-efficacy) were identified and discussed. These themes contributed to the formulation of SILVER, an innovative framework of components for consideration in EAP course design and delivery within higher education.

## 1. Introduction

The learning of English for academic purposes (EAP) and its impacts upon students’ academic studies have been substantially researched in English-medium universities, with most of the prior research focused on international students who speak English as a foreign language (EFL) [1,2,3,4]. Domestic students, however, have been under-served with EAP provisions as well as under-researched regarding the learning of their EAP, and its impacts upon their academic studies. These appear to result from the assumption that they would be naturally equipped with academic English skills since they speak English as their first language, an assumption that appears to be increasingly untenable [5]. In fact, domestic students have been reported to perceive difficulty in the use of academic English for university studies, and this is evident among those who are also multilingual speakers such as in the context of Singapore [6]. 

In recent years, certain EAP provisions have been implemented for domestic students on admission into autonomous universities in Singapore (i.e., universities under the ambit of the Singapore Ministry of Education), particularly for those who have not met the minimum English language requirement upon entry. This requirement is a Grade B4 in GCE O-Level English Language (i.e., Singapore-Cambridge General Certificate of Education Ordinary Level, recognised internationally as an equivalent to the General Certificate of Secondary Education [GCSE] examination in the United Kingdom). Despite this implementation, there has been a dearth of research to understand students’ learning experiences or the impacts of these EAP provisions. It is imperative to understand how domestic students experience these EAP provisions and their corresponding perceptions ranging from course design, content, to delivery, as these offer insights into how EAP provisions could better support student learning in the university. Particularly for the university in this study that had its revamped EAP programme implemented in 2017, and for the reasons discussed, it is worth exploring the learning experiences of students who offered the programme as well as its impacts on their subsequent academic studies in the university. Along with the findings of this study, a framework of components for consideration in EAP course design and delivery has been summarised, with the view that this framework may be applicable to other English-medium universities, especially those with a multilingual environment. 

## 2. Literature Review

This section presents the literature review undertaken as part of this study. Three major areas are discussed: (1) associations between EAP proficiency levels and academic performance in university (2) domestic students in Singapore and how English is their Lingua Franca, and (3) students’ learning experiences with EAP.

### 2.1. EAP Proficiency Levels and Academic Performance in University

English-medium universities have EAP requirements for admission of international students who are not L1 speakers of English given that EAP proficiency is an important condition for academic achievements. In addition to International English Language Testing System (IELTS) and the Test of English as a Foreign Language (TOEFL), higher education institutes have also been accepting other forms of EAP proficiency proofs such as locally developed tests [7,8], given that locally developed EAP proficiency tests might be advantageous because of their alignment with local curriculum [9]. 

Studies about the relationship between EAP proficiency scores and academic performance have been extensively conducted among international students, but findings have been inconclusive. Moderate correlations between IELTS/TOEFL scores and academic performance have been reported [10,11,12], as with weak correlations [1,13] and no correlations [14,15]. This inconclusive relationship between EAP proficiency scores and academic performance (usually indicated by Grade Point Average [GPA]) was also observed in prior research of some locally developed EAP tests. To exemplify, a significant correlation between an institutionally developed EAP proficiency test and students’ GPA was found [16], but an earlier study [17] contradicted this finding. 

Despite the inconclusive relationship in the prior research, it is worth mentioning that the literature has highlighted that international EFL students were generally found to obtain lower GPAs than their non-EFL counterparts (i.e., domestic students). This suggests the importance of EAP proficiency for academic success in English-medium universities [2,18]. Whether EAP proficiency plays a similar role in domestic students’ academic studies in English-medium universities remains unanswered, especially in those students who did not meet the minimum EAP requirement on admission. Notably, a recent study on domestic students in the United Kingdom pointed out the lack of EAP provisions for this group in higher education [5]. Nevertheless, the results of the study suggested that EAP provisions should target all students regardless of their linguistic backgrounds as domestic or international students. This has also been suggested by researchers who found that the acquired vocabulary size of home students in British universities was surprisingly low, and this could contribute to their struggle of academic studies even though these students speak English as their first language [19,20].

It is noteworthy that, based on the university in this study, an earlier quasi-experimental study found that EAP provisions had a statistically significant and positive intervention effect on the GPA of the first semester upon matriculation [21]. This earlier study provided information limited to intervention effects on GPA and hence, it is worth exploring students’ perceptions and learning experiences of EAP programmes in this study.

### 2.2. Domestic Students in Singapore: English as Lingua Franca in a Multilingual Context

In Singapore, English has been the lingua franca and predominant medium of instruction at all levels of education, but most Singaporeans are bilingual or multilingual speakers who might speak a different ethnic language (e.g., Hokkien, Teochew, Cantonese, and Hainanese) with family or friends [6,22]. Students’ struggle in academic English at different educational levels has been observed, and there is a rising number of studies researching the EAP proficiency of Singaporeans who speak English as the lingua franca in a multilingual context. Most of these studies have focused on the early stages of education [23,24,25] with far fewer research within tertiary education and hence, there is limited understanding regarding the impact of multilingual domestic students’ EAP proficiency levels upon their academic studies in university. 

A recent study investigating the multilingual ecology of Singapore suggested that domestic students do not consider themselves fully proficient in any of the languages they speak, including English due to the possibility of “a more language conscious perspective of multilingual speakers in comparison to monolingual speakers” [26]. This finding echoes a later study discovering that Singapore’s domestic students also perceived a struggle with EAP in their academic studies; teaching staff also agreed with the claim of insufficient EAP skills among domestic students [6]. This is unsurprising since the target students of EAP provisions have always been international students in English-medium universities, and domestic students have been left out with the assumption that they already have those skills. However, it has been pointed out that an increasing number of students, including L1 English speakers, have been admitted into university without sufficient academic communication skills, and this has made EAP proficiency a crucial aspect of their university learning [27]. In the same vein, it has been argued that students from all linguistic backgrounds should be supported with EAP provisions to facilitate their academic studies [28]. Evidently, more research is needed to understand the learning of EAP for domestic students in Singapore, and its impacts upon their academic studies in university, especially for students who were admitted into the university without fulfilling the minimum EAP proficiency requirement. 

### 2.3. Students’ Learning Experiences with EAP

Most of the prior research in EAP programmes focused on students’ subsequent performance in their EAP assessments and academic studies rather than learning experiences in the EAP programme [29,30,31,32]. Little research has provided insights into how students perceive the EAP programme, and whether they even understand the purpose of such a provision. 

Among the few studies exploring students’ perceptions and experiences in EAP programmes, the target group has been international EFL students. For instance, in comparing international postgraduate students who were required to complete the EAP course to those who had not taken the EAP course in an Australian university after admission, it was found that the former seemed to demonstrate better knowledge of study skills in reading and writing [33]. Students’ perceived experiences in listening and speaking, on the other hand, seemed similar across both groups. It is noteworthy that students enrolled in the EAP course found it useful to apply the skills acquired in the EAP course to their subsequent academic studies, especially the skills of reading comprehension, summarising texts and structuring essays. Those students also expressed greater satisfaction in their subsequent learning in the university compared with their counterparts who had not gone through any EAP training after admission into the university. The study, therefore, advocated for the expansion of EAP provisions to all the international students who would benefit in different ways to cope with their academic studies. As a development of this view, it has been argued that EAP support should be extended to native speakers of English who are usually wrongly seen to have the language skills for academic studies merely because they speak English as the first language; these students might actually need to develop EAP skills just as much as their peers who speak English as a second or foreign language [34]. 

Within tertiary education, research on EAP provisions has explored the effectiveness of the potential learning transfer from EAP training to students’ subsequent academic studies in their own disciplines. To exemplify, it was found that international students received EAP training attempted to transfer the skills acquired from EAP courses to their discipline learning, though the lack of support for the transfer did create some hurdles [35]. A recent study focusing on international students in an American university also found evidence of learning transfer as declared by students enrolled in an EAP writing course upon admission. In addition to the perceived holistic improvement in academic English skills, these students described the EAP writing courses as useful preparation for their own major courses in the university, so that they could more easily fulfil the requirement of their disciplinary studies [36]. 

On the other hand, other studies have argued that the learning transfer of EAP courses might not always take place. A longitudinal study with domestic students from an American university found that students claimed that they acquired valuable skills in the first-year composition course but did not perceive the application of these to other courses in university learning [37]. 

As shown in the literature review, most of the previous studies focused on the relationship between students’ EAP levels and their academic performance, especially among the international students who speak English as a foreign language. Notably, the results of those studies do not seem to be conclusive regarding the role of EAP in predicting students’ academic performance. Since the multilingual domestic students in English-medium universities are also facing the EAP challenges, there is a need to understand those students’ EAP learning experiences and how the EAP provisions can be improved to facilitate their academic studies in the university. More importantly, it is critical to understand how the students’ EAP learning experience can help their academic studies through the learning transfer of the EAP provisions, which has not been researched much in the previous studies.

## 3. Methodology

This study aimed to explore domestic multilingual students’ perceptions and learning experiences of an institutionally developed EAP programme. Specifically, the research questions for this study were: (1) what are students’ learning experiences of the institutionally developed EAP programme? (2) How do students’ learning experiences of the EAP programme impact their academic studies? A qualitative approach [38] was adopted for this study as it allowed for exploring the phenomenon via students’ perceptions and experiences in the programme. 

### 3.1. Research Participants

A maximum variation strategy [39] was used to identify a purposeful sample. The target population was domestic students who were admitted into university without fulfilling the minimum language proficiency requirement and hence, were enrolled into the EAP programme on admission. To be admitted into undergraduate programmes in Singaporean universities, all domestic students are expected to meet a language proficiency requirement. The typical proficiency requirement is Grade B4 in English Language of the GCE O-Level or its equivalents. Examples of its equivalents are Grade 4 in English for IB Diploma holders, a Grade B4 in English Paper at SPM (Sijil Pelajaran Malaysia, or Malaysian Certificate of Education, equivalent to the GCSE in England, Wales and Northern Ireland), a CAP (cumulative average point) of 3.0 in English Language for NUS (National University of Singapore) High School Diploma holders, an IELTS Academic score of 6.5, or a TOEFL score of 580 (paper-based), 237 (computer-based), or 85 (internet-based). 

The sampling criteria were as follows: (1) students who had successfully completed the EAP programme, (2) variety of demographic backgrounds based on age, gender and ethnicity, (3) variety of academic backgrounds in the university based on students’ academic discipline and their academic performance indicated by GPA at the time of data collection.

In total, 50 students across four academic faculties that the EAP programme is applicable agreed to participate in this study (see Appendix A).

### 3.2. Data Collection

Semi-structured interviews were used to collect data related to students’ perceptions and learning experiences within the EAP programme. An interview protocol (see Appendix B) was used as it afforded variation in the question order as well as potentially additional questions and probes to individuals [40]. Consistent with the main purpose of the study, interview protocol focused on participants’ language use, perceived challenges in academic English, factors that impact their academic English, and perceptions and learning experiences in the EAP programme. Each interview was conducted via Zoom, lasted between 45 to 60 min, recorded and transcribed. 

### 3.3. Data Analysis

Thematic analysis, widely used in qualitative research to develop patterns of meaning based on the dataset of texts, was used to extract the data [39]. In thematic analysis, codes would initially be developed on the descriptive level based on the raw data, and themes would be generated on the inferential level that could address the research questions. With the interview data from the present study, thematic analysis was suitable as patterns in relation to the research questions could be explored. An inductive approach was adopted for the thematic analysis where themes were generated based on the data. The data were coded and analysed using NVivo Software version 12 (QSR International Pty Ltd., Burlington, MA, USA). To ensure that the qualitative data were interpreted accurately; two research team members experienced in qualitative analysis reviewed the codes and themes thoroughly in relation to the research questions and literature. The members performed this independently before the research team gathered to validate the analyses, where differences in opinions in the development of codes and themes were discussed until a consensus was reached. Consistent data triangulation among the researchers was elemental to reducing the possibility of one researcher’s subjective perspective giving bias the interview analyses [41].

**Figure 1:**
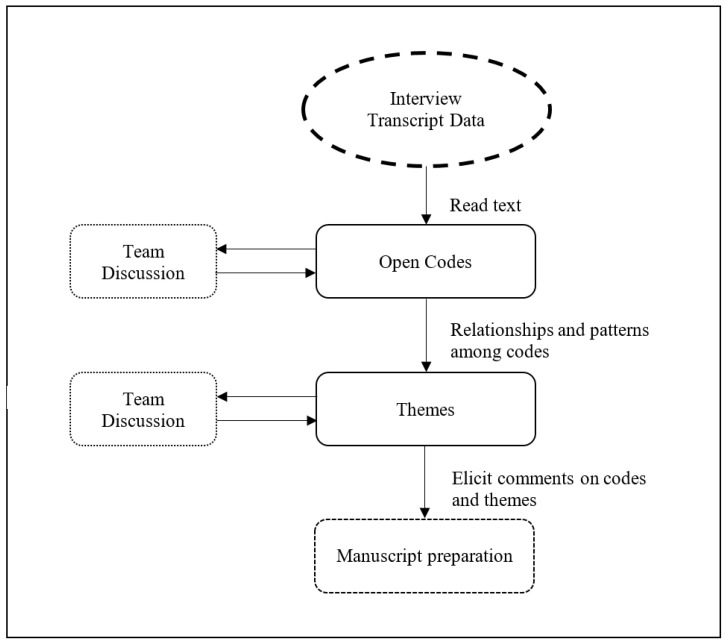
Flowchart of thematic analysis approach.

Figure 1 summarises the processes from coding to forming themes in this study.

**Figure 2:**
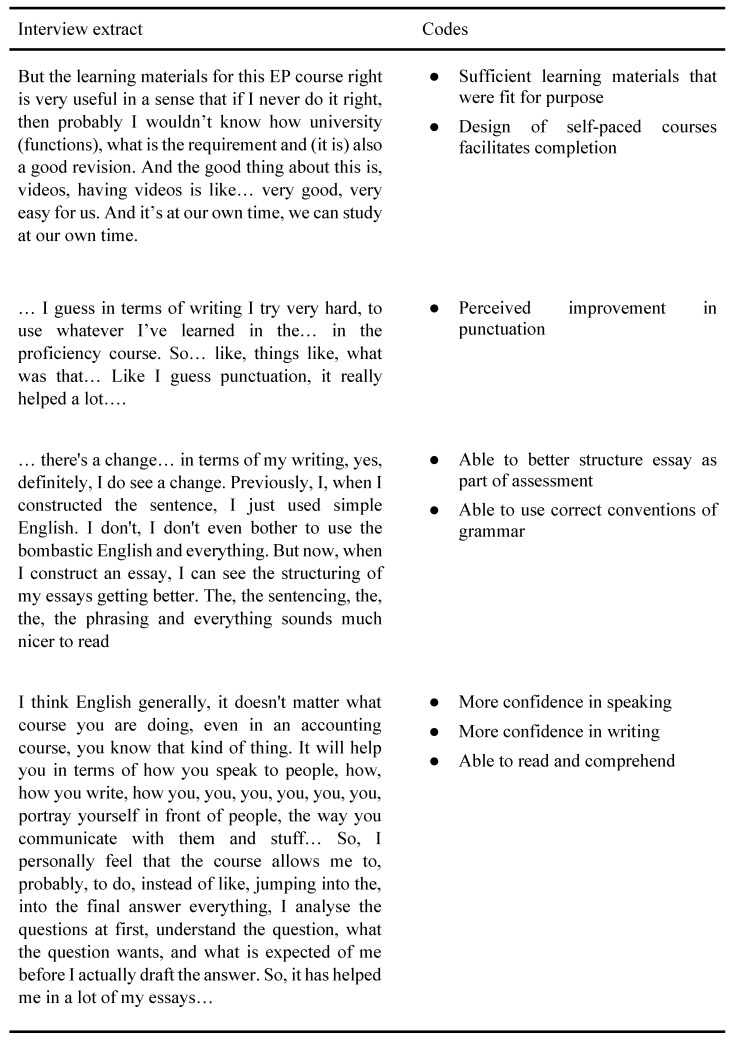
Example of coded interview transcript.

Following a thorough review of the interview transcripts for data familiarisation, the researchers applied codes that subsequently led to pattern exploration and the grouping of codes and themes (See Appendix C). As an example, codes derived from participants’ perceptions about developing better reading and academic writing skills were combined to form a meta-theme on improvement in English proficiency, leading to the overall theme on change in self-efficacy. Figure 2 provides an example of the coded interview transcript which subsequently guided the generation of themes.

## 4. Results

The thematic analyses undertaken based on the data collected suggested four themes. The following sections present the findings that inform the research aims (i.e., exploring student learning experiences of the EAP programme, and exploring the impact of these learning experiences on their academic studies) with the view of extending these findings to inform practice through contributing to the formulation of a framework. 

### 4.1. Overall Programme Perceptions

Slightly more than half the students interviewed (i.e., 26 of 50) expressed initial negative programme perceptions in that they were initially reluctant to enrol or they felt forced to complete the tasks within the EAP programme. As student HHES said:

So, I don’t believe just because we have a bad grade back in our O-Levels, it means that we have to go through with this English programme. Making it voluntary would, I guess, make us feel better to go through with it.

Student MHBK also expected the EAP programme to be time consuming and hence, stress students unnecessarily:

… if I’m in the business school… there is a lot that I need to complete, and the fact that I need to do the EP programme is like adding on to the stress and everything.

Of those who did not express an initial negative perception of the EAP programme, one had neutral perceptions of the EAP programme in that it was seen just as a graduating requirement. As student TKSD said:

For me, it was a task to complete… it was just, something to check and move on…

While most did not articulate initial positive programme perceptions such as how the programme would be able to diagnose students’ academic writing and hence, be useful for structuring and writing reports, student TSYS said:

… it will help students know more about, understand, have a better understanding of the university grading, as in like, the university expectations on the students’ command of English. For example, like reporting, report writing skills, those like presentation, all those areas…

Evidently, there were slightly more negative initial programme perceptions. The negative perceptions arose predominantly due to the view that the EAP programme was a forced task, given that completing and passing it are graduating requirements for students who did not meet the minimum EAP requirement upon matriculation. Despite this and the fact that EAP programme scores do not affect GPA, some of the interviewees expressed reluctance to enrol in the programme and questioned why they should even be enrolled to the programme. Further, since it is a mandatory requirement, some students shared that they were preoccupied with wanting to complete it within the shortest possible time and at the earliest opportunity.

Retrospective perceptions, beyond the initial ones mentioned above, of the EAP programme were generally mixed (i.e., positive and negative) though some expressed that they expected more of the programme. Negative perceptions were indicated by student perceptions that their English proficiency did not improve as a result of completing the EAP programme or that the programme did not meet their expectations; where students viewed that the EAP programme was useful in some areas was indicative of positive perceptions. Eight students, for instance, thought that the EAP programme was useful for report or essay writing; eight students said it was useful for learning citations and referencing; nine participants said it was useful for learning grammar, and nine participants said the EAP programme would be helpful for non-fresh school leavers (i.e., students who have been working for a period before university matriculation). A further review of the interviews of these 26 students uncovered a reflection of mismatched expectations of programme design or content; this could have explained why they thought the EAP programme did not meet their expectations. Some of the more telling mismatched expectations included a call for more instruction on citation formats, referencing, and a stronger connection between EAP and discipline-specific writing styles. As student CHM commented:

No, I don’t think (the programme) should be made compulsory… I think it’s more for let’s say like, citation wise, then I think they should like really have like, a proper lesson or module and make it compulsory for all students attend.

While these expectations seem to warrant that there is more to be achieved within the EAP programme, the university had designed a complementary academic integrity programme that is made known to all students upon matriculation, within which there is a module on proper citation and referencing. Further, 3 of the 26 students who had mixed perceptions expressed dissatisfaction about the EAP programme not fully meeting their expectations related to the feedback provided throughout the full online programme. These students had hoped for more guidance on their academic writing weaknesses or wanted more personalised feedback.

#### 4.1.1. Programme Delivery

Programme delivery in this study comprised how the programme was delivered and taught to the students, and the adequacy of learning content within the programme. Most of the students interviewed articulated their perception that the EAP programme was appropriately designed and delivered and that there were sufficient learning materials. This is reflected by student NGYA:

But the learning materials for this EP course, right, is very useful in a sense that if I never do it right, then probably I wouldn’t know how university (functions), what is the requirement and (it is) also a good revision. And the good thing about this is, videos, having videos is like… very good, very easy for us. And it’s at our own time, we can study at our own time.

In addition, student IMFM commented:

Yeah, personally, I feel like it is quite helpful. Like, there is a lot of detail, yeah, it is quite detailed, that is very important. And it is like, when you go through the learning materials, right, they really provide you everything like, from scratch like, all those basic information which is, personally, I feel it’s very useful.

As can be seen, designed as a fully online and self-paced programme where students would have minimum interactions with human instructors, students felt that the EAP programme design and structure paced their learning appropriately. It is noteworthy that nine of the students interviewed expressed that the EAP programme delivery mode and how it was intended to be self-paced facilitated their completion (of the programme) particularly when they had to manage other commitments such as work and family.

#### 4.1.2. Linguistic Improvement

The perceived appropriate programme delivery appeared to be consistent with perceived improved linguistic skills. To illustrate an improvement in a topic within the EAP programme, punctuation, student PLW said:

… I guess in terms of writing I try very hard, to use whatever I’ve learned in the… in the proficiency course. So… like, things like, what was that… Like I guess punctuation, it really helped a lot….

In another example, student LYX commented on an improvement in grammar:

I think it helps me actually quite a lot in like writing, yeah, in writing. I think I used to… was not aware that my grammar was wrong and then I do not even like further check it. But for now, I actually, like after writing everything, I will check all the sentences.

In terms of improvement in paraphrasing, MHBK said:

Paraphrasing… paraphrasing used to be hard for me last time. Like, I always don’t understand how it was done and everything. But I think the EP programme like taught me like, okay, there are other ways of paraphrasing your, your answers. You don’t necessarily have to be like that, but it can be like this. And, but what is important is the gist and the summary of what you are trying to paraphrase there.

Of the 50 students, 35 felt they had improved linguistic skills (e.g., grammar, punctuation, paraphrasing). The remaining 15 felt they had little or no improvement to their linguistic skills upon completing the programme. As highlighted earlier, this could be attributed to a mismatch of expectations, as they expected the programme to focus more on citation and referencing, and they assumed that citation is a linguistic skill.

#### 4.1.3. Learning Transfer

Beyond the programme perceptions related to learning experiences, practically all the students interviewed expressed that they were able to apply what they learnt out of the EAP programme to their subsequent academic studies. This is most evident out of what student MHBK said:

… there’s a change… in terms of my writing, yes, definitely, I do see a change. Previously, I, when I constructed the sentence, I just used simple English. I don’t, I don’t even bother to use the bombastic English and everything. But now, when I construct an essay, I can see the structuring of my essays getting better.

Alongside the majority of students who expressed that they were able to apply what they learnt from the EAP programme to subsequent academic studies, remarkably, this observation also applied to the minority that had some mismatched expectations of the programme. The learning transfer and application were observed as one or more of the following forms: (1) able to use correct conventions of grammar (2) able to better structure essays (3) able to write with varying styles to suit requirements of different courses (4) able to build arguments (5) able to read and comprehend and, (6) able to do proper citation and referencing.

#### 4.1.4. Changes in Self-Efficacy

One of the objectives of the EAP programme is to enhance English proficiency by developing better reading and academic writing skills. It is expected that with enhanced EAP skills, students would be more confident and better supported in their academic pursuits. This expectation is not unfounded, as a separate study found that students who completed the same EAP programme in the university had better than predicted GPA [21], suggesting that English proficiency has a positive impact on GPA. In this study, 24 of 50 students interviewed articulated that completing and passing the EAP programme helped with improving their English proficiency. Of these 24 students, 4 specifically said that the EAP programme provided them with a confidence boost. For example, Student LCKM said:

Then… for me to be able to complete the EP (English proficiency) programme, I was a bit more relieved, and I think that also gave a little bit more confidence because, I would assume that the EP programme is done by the English professors and all. So, if at their level it’s acceptable, then, it gave me some confidence to continue writing for the other modules as well.

The remaining students felt that their English proficiency was enhanced a little or not at all despite alluding to the ability to transfer and apply what they learnt from the EAP programme. Most from this group presented a mismatched expectation, as they expected the programme to be an instructional programme on proper citation and referencing, as reflected by what student MKA said:

I would say (my English proficiency remained) about probably the same. Around there… Probably minor (change).

Four themes arose from analysing the overall programme perceptions: (1) programme delivery (2) linguistic improvement (3) learning transfer and, (4) changes in self-efficacy. These themes provided information with regards to students’ learning experiences of the EAP programme, and how these experiences impacted their academic studies. Taken together, the four themes presented appear progressive based on the view that appropriate programme delivery can impact and improve linguistic skills; with improved linguistic skills, students would be better able to apply them in their academic studies and hence, learning transfer occurs, leading to a change in self-efficacy. 

Further to addressing the research aims, these themes have been extended to contribute to the formulation of a framework of components for consideration in EAP course design and delivery. This framework, SILVER (**S**pecification of purposes, **I**ntegration of progress measures, **L**ink of EAP content with disciplinary study, **V**aried formats of content, **E**xtended time of delivery, and **R**einforcement of selected topics) is elaborated in the following section.

## 5. Discussion

The current literature has been focused mostly on international students as the target group of EAP programmes. Along with reported student learning challenges after completing EAP programmes, evaluation studies have often tried to suggest the revision of programme content to promote student academic development [42,43,44]. Evident from this study, content does not seem to be the only obstacle of students’ progress in EAP skills or their subsequent academic studies in university. Other factors also need to be taken into account to promote linguistically weaker students’ academic progress across their entire candidature in university. More importantly, domestic students of English-medium universities have been mistakenly assumed to have automatically mastered EAP skills for their academic studies, which calls for immediate attention to extend EAP provisions to them [5,34]. 

Based on an extension of the findings of this study, a framework of components for consideration in EAP course design and delivery, SILVER, is proposed.

### 5.1. Specification of Purposes

As the findings show, more than half of the students interviewed had little understanding about the purpose of the provision, and primarily saw it as a punitive measure. As a result, they perceived the learning activities as a forced task, which led to their passive learning behaviours in the programme. This finding echoes the perception of international students enrolled into EAP programmes of English-medium universities [42]. Since students were often requested to complete the programme as part of their admission conditions, this extra workload was rarely perceived as a learning support as it was intended. Even the few students who tried to make use of this learning opportunity demonstrated mismatched expectations for the programme, which naturally gave rise to some of their negative learning experiences. Thus, it is essential to communicate specific purposes of EAP programmes to students who are required to take such provisions. This would help steer their expectations and prepare them for better usage of the learning support by going through the programme. 

### 5.2. Integration of Progress Measures

Findings from this study indicated that some students doubted their linguistic improvement despite completing the EAP programme. This suggests merit in integrating progress measures (e.g., testlets) within the EAP programme, as these could provide insights into students’ progress across time, along with multiple sources of evidence (e.g., the number of grammatical errors in assignments submitted for discipline-specific courses). An integration of progress measures could also help students with lower self-efficacy upon enrolment as they could track their learning progression and make adjustments that could impact their self-efficacy in EAP skills positively. In fact, students who perceived to have acquired useful skills in the EAP programme and increased their self-efficacy were found to express greater satisfaction in their subsequent learning in the university [33,34].

### 5.3. Link of EAP Content with Disciplinary Study

A common phenomenon among participants in this study was that they would try to link what they had learnt in the EAP programme with what they would be facing in their disciplinary study. Drawing from the progressive nature of the findings, linguistic improvement appears to be the first step, and learning transfer from the EAP programme to students’ disciplinary programmes is their ultimate goal. Prior research with international students also pointed out that future directions of EAP programmes should focus not only on general language development but also how academic English is expected in various disciplines [45]. Arguably, such a direction is also essential for EAP programmes offered to domestic students in English-medium universities since their linguistic levels are relatively higher than the international students, and their demand for learning transfer into their degree programmes could be stronger. 

### 5.4. Varied Formats of Content

Since the researched EAP programme is fully online, the lack of face-to-face instruction would require a variety of formats of the content in order to engage students across time. This is consistent with previous studies regarding the design of any full online course [46]. Findings from this study related to programme delivery indicated that students were satisfied and demonstrate the affordances of videos and plain text as part of the content in the EAP programme. Despite this, it is arguable that more interactive formats could be afforded. As [47] stated, what matters more is not one particular format that would automatically promote student engagement but the inclusion of various formats as a whole. 

### 5.5. Extended Time of Delivery

Since the EAP programme is an extra workload for students who did not fulfil the language requirement for admission, it is critical to facilitate their progress and completion by extending the time of delivery. Almost all of the participants in this study mentioned the benefit of starting the EAP programme earlier than their degree programme, which substantially contributed to their completion of this extra workload. Based on the extensive research of Post-entry English Language Assessment (PELA) in Australian universities, most of those provisions are offered during the semester when students have already started their own degree programmes [48,49]; this might hinder students’ engagement in PELA provisions. Different from the design of most PELA provisions, the current study adopted an earlier timeline in the EAP programme for each semester, so that students who are required to are given the chance to complete it before they start their disciplinary studies. 

### 5.6. Reinforcement of Selected Topics

Most of the students who successfully completed the EAP programme perceived minor to moderate progress of academic English, though they also realised the need to continue the effort of EAP development to fulfil academic expectations in their disciplinary studies. The reported challenges of academic studies after completing EAP programmes are common among international students, particularly in the aspects of understanding academic articles in their disciplinary study, or conducting more complex written assignments [33,50,51,52]. Hence, it is unsurprising to observe the same issue among domestic students who just completed the EAP programme. Despite perceived learning transfer, there remains a gap between what students have acquired from EAP programme and what they are expected to deliver in their degree programmes. This observation is more evident for students in disciplines that are more linguistically demanding. These suggest merit to continue EAP support for those students with targeted reinforcement of certain topics, such as constructing academic papers at a more advanced level and comprehending complex academic articles. This source of EAP support is necessary because students have difficulty in obtaining it from their own degree programmes, since the instructors are usually just the subject matter experts in the disciplinary areas who might also lack sufficient EAP skills, a phenomenon discovered in prior research [35].

## 6. Conclusions

This study sought to explore student learning experiences of an institutionally developed EAP programme, and the impact of these learning experiences on their academic studies. The analyses reported a progressive nature of themes generated from the interview data (i.e., programme delivery, linguistic improvement learning transfer, change in self-efficacy). Findings also suggest that domestic students of English-medium universities could be better supported in terms of EAP support in order to facilitate their academic studies in their degree programmes. While most students demonstrated positive perceptions and experiences in the EAP programme, some seemed to lack the understanding of the purpose of such provisions, and these mismatched expectations have led to negative attitudes as well as learning experiences in the university. As an extension to inform practice, the themes discussed contributed to the formulation of SILVER, a framework of components for consideration in EAP course design and delivery.

It is recommended that all components in the proposed SILVER framework be considered for effective curriculum design of EAP programmes and a productive learning experience for the students. While prior research emphasised the revision of programme content [43,44], the current study pointed out the necessity to consider other factors as specified in the SILVER framework.

Some limitations of the study should be noted. To begin with, the current study investigated students’ learning experience shortly after completing the EAP programme without tracking their developments across semesters. Future research could include longitudinal studies to understand longer-term impacts of the EAP programme upon the students’ academic studies in the university. Further, the coverage of academic subjects could be expanded beyond the four faculties involved for future research. The academic demands of different subjects could also be investigated as these might contribute to different student learning experiences.

## Figures and Tables

**Figure A1:**
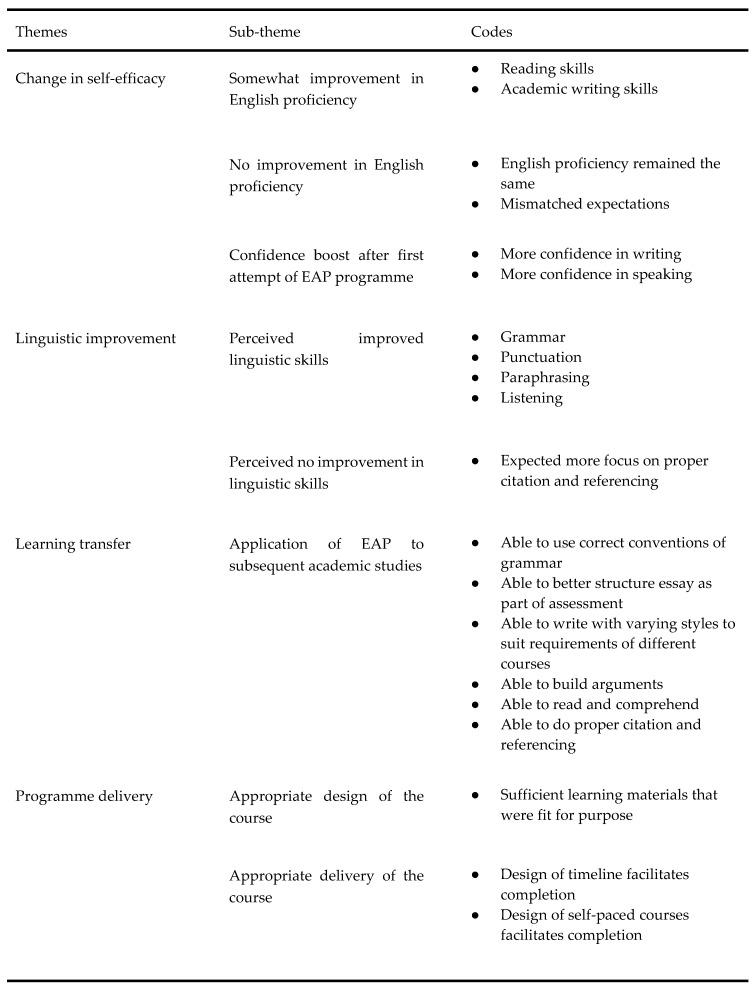
Summary of themes, sub-themes, and codes.

## Data Availability

Data available on request due to privacy and ethical restrictions.

## Appendix A

**Table A1:** Participant Profile.

Participant	Age	Ethnicity	Gender	Academic Discipline
JP	32	Chinese	Female	Business
PLW	23	Chinese	Female	Business
LCKM	23	Chinese	Male	Humanities and Behavioural Sciences
NRK	30	Indian	Male	Science and Technology
SAS	30	Indian	Male	Science and Technology
GKWJ	35	Chinese	Male	Science and Technology
TZL	25	Chinese	Female	Human Development
NGYA	36	Chinese	Male	Human Development
BTSL	42	Chinese	Female	Humanities and Behavioural Sciences
MKA	26	Malay	Male	Science and Technology
WMF	30	Chinese	Female	Science and Technology
TKSD	40	Chinese	Male	Human Development
SK	47	Indian	Female	Human Development
TBSS	43	Chinese	Male	Science and Technology
HHES	23	Chinese	Male	Business
CHM	22	Chinese	Female	Humanities and Behavioural Sciences
MHBK	31	Malay	Male	Business
TSYS	23	Chinese	Female	Business
CFPP	56	Chinese	Female	Humanities and Behavioural Sciences
AKPL	24	Chinese	Male	Human Development
DCEL	23	Chinese	Female	Humanities and Behavioural Sciences
VLQH	25	Chinese	Female	Human Development
LCYA	31	Chinese	Male	Science and Technology
LC	26	Chinese	Male	Business
SABK	26	Malay	Female	Human Development
SHBS	31	Malay	Female	Business
HBI	26	Malay	Female	Science and Technology
KYSJ	26	Chinese	Male	Human Development
SABS	35	Malay	Female	Human Development
LYX	23	Chinese	Female	Business
LJC	23	Chinese	Male	Business
AHAS	31	Malay	Male	Science and Technology
MHMS	27	Malay	Male	Science and Technology
DCCT	26	Chinese	Female	Business
NBMF	30	Malay	Female	Business
NSAL	24	Malay	Female	Science and Technology
CQ	23	Chinese	Female	Business
JYJL	23	Chinese	Male	Humanities and Behavioural Sciences
LJJ	28	Chinese	Male	Science and Technology
CMH	44	Chinese	Female	Human Development
JTW	26	Chinese	Male	Science and Technology
BTKW	25	Chinese	Male	Science and Technology
TYS	45	Chinese	Male	Business
LJ	29	Chinese	Male	Science and Technology
IMFM	28	Malay	Female	Business
TGL	33	Chinese	Male	Business
TL	48	Chinese	Female	Human Development
CCC	48	Chinese	Female	Humanities and Behavioural Sciences
SMSA	22	Others	Male	Science and Technology
NBA	48	Malay	Female	Humanities and Behavioural Sciences

## Appendix B

Semi-structured interview questions:

1. Describe your previous English learning experience
  - How did you prepare for high stakes English language exams previously e.g., PSLE/N/O/A/IB?
  - Did you have to engage additional help beyond school e.g., tuition, and why?
  - What were some classroom interactions that were memorable and useful for building your English proficiency e.g., class presentations/debates?
2. What language do you speak at home mainly (in % on a daily basis) and at work? How was this language used?
  - Do you use this language for communication/writing notes? How about English?
  - With whom do you use English with e.g., friends, and how much (% on a daily basis)?
3. How would you describe your English proficiency currently?
  - Do you face any issues where English is used in university e.g., understanding course content, assignment requirements?
  - How would you say your listening/reading/writing/grammar is? Is it within your expectations?
  - What were some factors that contributed to your current English proficiency?
  - Would you share whether your EP has improved since you were matriculated and how it has improved? What were some factors that helped (or hindered) in this improvement?
  - Have you been frustrated while having to complete assignments due to English proficiency?
  - How confident have you been (after completing the EP programme) in presenting in front of classes/completing writing assignments?
4. Currently, how much writing in English do you do (% on a daily basis) and for what purposes?
  - Did you use English for informal (IG/Twitter/Blogs), formal (class assignments, presentations)?
  - What was the frequency e.g., often, seldom?
  - Was informal/formal use of English in (a) useful in building your English proficiency?
5. What types of writing have you done so far in the university e.g., assignments? What is the length of the assignments (number of words)? Have you always exceeded the word limit? Describe how English proficiency impacted you in completing these assignments.
  - Did you experience difficulties with writing these assignments where language was a concern?
  - Have any instructors given you feedback on your writing or presentation with regard to articulation of ideas and English proficiency? How did you react to the feedback and what did you do about it?
  - Have you been worried about doing well for assignments due to English proficiency?
  - Have you had (and how often) concerns about assignment deadlines owing to English proficiency?
6. You would have completed the EP programme in the university. How has the EP programme helped you?
  - How is your learning experience in the EP programme in general?
  - What SDE courses (SDE101 diagnostic course, SDE102 basic writing course, SDE103 grammar, SDE104 academic reading, SDE105 advance writing course) were you enrolled? How was your learning experience in those courses, respectively?
  - What would you say has been most useful of the programme (and how it has been useful)?
  - What you would say is useful to have for students offering subsequent EP programmes? How would these be useful for students?

## Appendix D

**Table A2:** Summary table on the number of cases for each code.

Themes.	Sub-Theme	Codes	No. of Cases
Programme delivery	Appropriate design of the course	Sufficient learning materials that were fit for purpose	5
	Appropriate delivery of the course	Design of timeline facilitates completion	8
		Design of self-paced courses facilitates completion	9
Linguistic improvement	Perceived improved linguistic skills	Grammar	22
		Punctuation	6
		Paraphrasing	5
		Listening	9
	Perceived no improvement in linguistic skills	Expected more focus on proper citation and referencing	15
Learning transfer	Application of EAP to subsequent academic studies	Able to use correct conventions of grammar	8
		Able to better structure essay as part of assessment	15
		Able to write with varying styles to suit requirements of different courses	13
		Able to build arguments	10
		Able to read and comprehend	5
		Able to do proper citation and referencing	5
Change in self-efficacy	Somewhat improvement in English proficiency	Reading skills	6
		Academic writing skills	9
	No improvement in English proficiency	English proficiency remained the same	11
		Mismatched expectations	6
	Confidence boost after first attempt of EAP programme	More confidence in writing	6
		More confidence in speaking	1
